# Emotion Regulation and Excess Weight: Impaired Affective Processing Characterized by Dysfunctional Insula Activation and Connectivity

**DOI:** 10.1371/journal.pone.0152150

**Published:** 2016-03-22

**Authors:** Trevor Steward, Maria Picó-Pérez, Fernanda Mata, Ignacio Martínez-Zalacaín, Marta Cano, Oren Contreras-Rodríguez, Fernando Fernández-Aranda, Murat Yucel, Carles Soriano-Mas, Antonio Verdejo-García

**Affiliations:** 1 Department of Psychiatry, Bellvitge University Hospital-IDIBELL, Barcelona, Spain; 2 Department of Clinical Sciences, School of Medicine, University of Barcelona, Barcelona, Spain; 3 School of Psychological Sciences and Monash Institute of Cognitive and Clinical Neurosciences, Monash University, Melbourne, Australia; 4 CIBER Salud Mental (CIBERsam), Instituto Salud Carlos III (ISCIII), Barcelona, Spain; 5 Department of Psychobiology and Methodology in Health Sciences, Universitat Autònoma de Barcelona, Barcelona, Spain; 6 CIBER Fisiopatología de la Obesidad y Nutrición (CIBERObn), Instituto Salud Carlos III (ISCIII), Barcelona, Spain; Southwest University, CHINA

## Abstract

Emotion-regulation strategies are understood to influence food intake. This study examined the neurophysiological underpinnings of negative emotion processing and emotion regulation in individuals with excess weight compared to normal-weight controls. Fifteen participants with excess-weight (body mass index >25) and sixteen normal-weight controls (body mass index 18–25) performed an emotion-regulation task during functional magnetic resonance imaging. Participants were exposed to 24 negative affective or neutral pictures that they were instructed to Observe (neutral pictures), Maintain (sustain the emotion elicited by negative pictures) or Regulate (down-regulate the emotion provoked by negative pictures through previously trained reappraisal techniques). When instructed to regulate negative emotions by means of cognitive reappraisal, participants with excess weight displayed persistently heightened activation in the right anterior insula. Decreased responsivity was also found in right anterior insula, the orbitofrontal cortex and cerebellum during negative emotion experience in participants with excess weight. Psycho-physiological interaction analyses showed that excess-weight participants had decreased negative functional coupling between the right anterior insula and the right dlPFC, and the bilateral dmPFC during cognitive reappraisal. Our findings support contentions that excess weight is linked to an abnormal pattern of neural activation and connectivity during the experience and regulation of negative emotions, with the insula playing a key role in these alterations. We posit that ineffective regulation of emotional states contributes to the acquisition and preservation of excess weight.

## Introduction

Worldwide, the number of people with excess weight has increased from 857 million in 1980, to 2.1 billion in 2013 [1]. The increase in incidence of overweight and obesity is particularly high during young adulthood [2] and is of concern given that earlier onset of obesity is associated with poorer recovery and outcomes [3]. Interventions that solely focus on correcting the imbalance between caloric intake and energy expenditure have proved to be ineffective and are frequently characterized by momentary weight loss followed by weight gain once treatment has ended [4], indicating the presence of underlying maintenance factors. To this end, a growing body of evidence has established a robust link between negative affect, coping styles and increased weight [5].

It has been suggested that chronic negative affective states result in the acquisition of maladaptive coping strategies such as the excess intake of appetizing and unhealthy foods to suppress negative emotions [6]. An indirect causality model is supported by evidence showing that individuals with high body mass index (BMI) show a stronger association between chronic stress and weight gain compared to low-BMI individuals who experience stress to a similar extent [7]. In addition, negative moods have been found to potentiate brain response to food [8] by influencing mesolimbic circuitry and reward-seeking behavior [9]. More specifically, imaging studies have linked obesity to a reduction of dopamine striatal D2 receptors [10], and to decreased prefrontal metabolism in the ortbitofrontal cortex (OFC) and anterior cingulate cortex (ACC) [11]. Other research, in a wide range of different populations, has found that dysfunction in these two aforementioned regions results in alterations in salience attribution and inhibitory control, respectively [12,13]. Overall, this suggests that an inability to properly value and manage negative affective states could be a key aspect in the acquisition and maintenance of excess weight as those who are unable to manage negative states might be more vulnerable to becoming stressed and consequently seeking relief in the rewarding properties of highly palatable foods [14]. Furthermore, weight-based discrimination is highly prevalent in society [15] and has been positively associated with negative attitudes towards weight problems and low self-esteem, thus further aggravating negative mood states and encouraging harmful food intake in these populations [16].

To date there is a paucity of studies exploring emotion processing and regulation networks in people with excess weight. However, researchers have found that the neural systems involved in managing emotions and in regulating appetitive responses to palatable foods highly overlap [17,18]. As the presence of stressors and negative emotions are inevitable facets of everyday life, the way one regulates negative affect can shape eating behavior [19]. Emotion regulation refers to the set of distinct, goal-based strategies by which individuals attempt to control how they experience (i.e., the nature, timing and reactivity to) these emotions [20]. Typically, the most studied emotion regulation strategy in brain-imaging studies has been cognitive reappraisal, which involves reinterpreting the meaning of affective stimuli in order to modify their emotional intensity [21]. During reappraisal, prefrontal control systems are understood to modulate activity in affect systems as a function of one’s regulatory goals and the nature of emotions being regulated [22]. Specifically, the dorsolateral prefrontal cortex (dlPFC) is known to support the manipulations and cognitive demands of appraisals via working memory whereas the ventrolateral prefrontal cortex (vlPFC) involves the selection and/or inhibition of appraisal resources [23–25]. Furthermore, the dorsomedial prefrontal cortex (dmPFC) assists in monitoring and providing the meaning of changing emotional states while the ventromedial prefrontal cortex (vmPFC) modulates activity in regions linked to emotional responses, such as the amygdala and the insula [26–28].

Reappraisal has been so widely studied due to the fact that its cognitive and physiological costs are lower than other emotion-regulation strategies (e.g. expressive suppression and distraction) and that it is highly effective at regulating affect and physiological arousal [29,30]. Indeed, the use of ineffective or maladaptive emotion-regulation strategies, such as expressive suppression [31], over adaptive strategies like reappraisal has been found to lead to increased food intake [32]. However, no studies to date have explored the neural substrates of emotion regulation by means of cognitive reappraisal in people with excess weight.

In this study, we used functional magnetic resonance imaging (fMRI) to compare the brain substrates of emotion regulation in excess-weight individuals versus normal-weight controls. A modified version of the original cognitive reappraisal task designed by Phan et al. [33] was used to assess emotion regulation. This task [34] has been shown to activate the brain networks involved in the generation of a negative emotional state [21,35–37] (i.e., insula and amygdala), as well as in the employment of cognitive emotion-regulation strategies (i.e., prefrontal cortex) [22,23,38,39]. To further explore the interaction between emotion generation and emotion regulation systems, we also performed a psycho-physiological interaction (PPI) analysis and assessed changes in the connectivity between these two systems across emotion regulation and generation blocks.

On the basis of previous literature [9,22] we hypothesized that excess-weight participants would exhibit heightened reactivity indicated by altered activation in emotion-generation and interoception-related regions when viewing negative stimuli compared to normal-weight controls. Additionally, we expected excess-weight participants to display inefficient down-regulation of negative emotional responses during reappraisal, and that this would coincide with persistently heightened activation in emotion-generation areas and reduced activity in emotion-regulation areas. Moreover, since emotion regulation [40], impulsivity [41], approach/avoidance motivation [42] and food addiction [43] are key trait and state characteristics that contribute to the acquisition and maintenance of excess weight and related affective alterations, and in agreement with theoretical accounts about the associations between these factors [44], we expected to find a complex interaction between behavioral measure results assessing these traits and areas of dysfunctional activation in the excess-weight group. These complex associations were subsequently explored by means of path analysis.

## Methods

### Participants

Participants were recruited through community notice boards at Monash University, Melbourne, Australia. Potential participants were invited to complete an online survey that assessed their eligibility to participate in a neuroimaging study on emotions and weight. Written and signed informed consent was obtained from all subjects after they were provided with a complete description of the study, which was reviewed and approved by the Monash University Human Research Ethics Committee (approval number CF13/2459–2013001307).

Young adults from both sexes were eligible for the study if they met the following inclusion criteria: (i) aged between 18 and 24; (ii) BMI ranges between 18 and 40 (people with a BMI higher than 40 were excluded from the study due to potential confounding metabolic variables). BMI was calculated from measurements of height and weight obtained using standardized procedures and equipment (Seca 700 Mechanical Column Scale) at the Monash Biomedical Imaging Centre (MBI), and calculated using the following formula: weight (kg)/ height (m^2^). Participants with a BMI between 18 and 25 were placed in the normal-weight group and participants with a BMI greater than 25 were placed in the excess-weight group. The exclusion criteria were: (i) current comorbid medical conditions associated with excess weight (e.g., type II diabetes, hypertension); (ii) history of a psychiatric disorder or current psychiatric symptoms (e.g., depression); (iii) history of head trauma or neurological illness involving the central nervous system; and (iv) MRI contraindications (e.g. piercings, etc.).

Fifteen excess-weight participants and sixteen normal-weight controls participated in the study. Results from two participants (one normal-weight control and one excess-weight participant) were excluded due to excessive movement during image acquisition, as were the results from an additional normal-weight participant for failure to carry out the reappraisal task. The final sample thus consisted of 14 excess-weight subjects and 14 normal-weight controls (6 males and 8 females for both groups). Demographic and behavioral data from the study participants are summarized in Table 1.

**Table 1:** Sample characteristics and behavioral measure results.

	Excess weight	Normal weight	Statistic
	(n = 14)	(n = 14)	*(p-value)*
**Age**	21.71 ± 1.81	21.21 ± 1.42	**-0.811 (.425)**
**Male/female**	6/8	6/8	**χ² = 0.00 (1)**
**Weight (kg)**	90.03 ± 15.44	60.79 ± 9.36	**-6.059 (0.000)**
**BMI**	31.85 ± 4.70	20.94 ± 1.64	**-8.194 (0.000)**
**Height (m)**	1.68 ± 0.09	1.70 ± 0.09	**0.517 (0.610)**
Education Level*	5.43 ± 0.85	5.07 ± 0.28	**χ² = 4.16 (0.125)**
Emotion Regulation Questionnaire (ERQ)**			
**Cognitive reappraisal score**	22.54 ± 7.49	23.71 ± 5.62	**0.464 (0.188)**
**Expressive suppression score**	18.77 ± 4.97	21.07 ± 4.25	**1.297 (0.194)**
**Barratt Impulsiveness Scale (BIS-11)**			
**Total score**	47.29 ± 11.23	37.79 ± 5.25	**-2.868 (0.008)**
**Attentional**	16.79 ± 3.64	12.50 ± 2.65	**-3.559 (0.001)**
**Motor**	20.64 ± 5.108	19.57 ± 3.005	**-0.676 (0.505)**
**Non-planning**	9.86 ± 4.294	5.71± 3.197	**-2.896 (0.008)**
**Yale Food Addiction Scale (YFAS)**			
**YFAS symptom count**	2.71 ± 1.94	1.93 ± 0.99	**-1.349 (0.189)**
**The Behavioral Inhibition System and Behavioral Activation System**			
**Behavioral Inhibition System score**	39.14 ± 5.947	38.93 ± 4.938	**-0.104 (0.918)**
**Behavioral Activation System score**	17.71 ± 2.585	16.71 ± 2.091	**-1.125 (0.271)**

Data are means ±SD;

* 1: No formal education 2: Did not complete high school 3: Completed high school 4: Diploma 5: Undergraduate degree/currently completing 6: Undergraduate degree/completed 7: Postgraduate degree/currently completing 8: Postgraduate completed

** Excess weight n = 13 *statistic* = t-values unless otherwise indicated.

### Procedures

Eligible participants were scheduled for image acquisition after a light meal (breakfast or lunch), between 8 a.m. and 2 p.m., at the Monash Biomedical Imaging facility (MBI). Participants were weighed and measured and briefly interviewed about their medical history to confirm they met inclusion criteria. Participants were given task instructions ahead of the scanner session and trained to decrease the intensity of their emotions through cognitive reappraisal techniques such as distancing and reinterpretation [34]. After the general training on reappraisal techniques, all participants performed a brief rehearsal of maintenance and reappraisal strategies using trial images. Only after successful rehearsal were the participants entered into the scanner.

### fMRI task: cognitive reappraisal task

**Fig 1:**
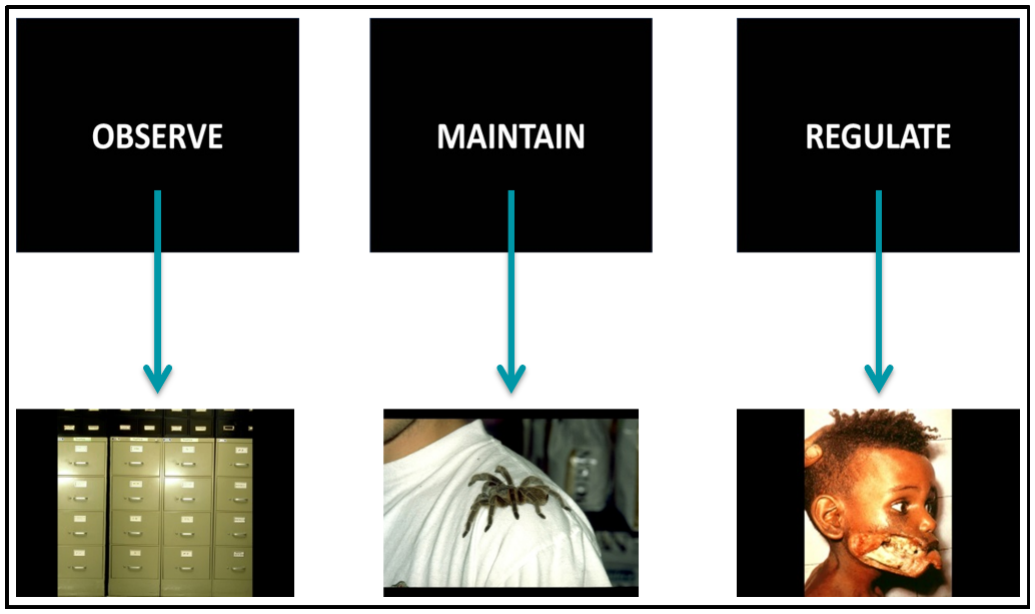
fMRI task. Example images for Observe, Maintain and Regulate conditions.

We used a modified version of the original cognitive reappraisal task designed by Phan et al. [33]. This task has been used to study emotion regulation in different psychiatric populations as well as in healthy controls [21,34]. The task consisted of presenting a series of blocks displaying neutral or negative picture stimuli that participants were instructed to (1) Observe (to passively observe neutral pictures); (2) Maintain (to actively pay attention to the emotions elicited by negative emotional pictures, sustaining them over time); or (3) Regulate (to reappraise the emotions induced by the negative emotional pictures by means of previously trained cognitive reappraisal techniques). Task instructions and visual stimuli were presented through an MRI-compatible angled mirror system and using Presentation^®^ software (Version 0.70, www.neurobs.com) (see Fig 1).

We used 24 stimuli that were extracted from the International Affective Picture System [45]: eight neutral pictures (e.g. household objects), which were presented in the Observe condition and 16 highly unpleasant, arousing pictures (e.g. mutilations) in the Maintain and Regulate conditions. Specifically, it consisted of 12 blocks: four blocks for each condition. Instructions (Observe, Maintain or Regulate) were pseudo-randomized throughout the task to avoid the induction of sustained mood states. Each block began with the instructive prompt (Observe, Maintain or Regulate) presented in the middle of the screen for 4 seconds. After the prompt, participants viewed two different pictures of equal valence for 10 seconds each. After the presentation of the second picture of each block, the intensity of the negative emotion experienced was self-rated by participants on a 1–5 number scale (1 being ‘neutral’ and 5 being ‘extremely negative’). These in-scanner ratings were recorded through an fMRI-compatible response pad (Lumina–Cedrus Corporation).

Each block was followed by 10 seconds of baseline during which a cross fixation was presented on the screen to minimize carryover effects. Further descriptions of our task procedures have been reported elsewhere [34].

#### Stimuli

The images were selected according to International Affective Picture System normative values for valence and arousal [45]; mean valence values were 5.79 (0.71), 2.53 (0.69), 2.66 (0.68) and mean arousal values were 4.28 (0.73), 6.44 (0.46) and, 6.40 (0.60) for images included in the Observe, Maintain, and Regulate conditions, respectively. Pairwise comparisons showed that Maintain and Regulate did not differ between them in valence or arousal (P > 0.7), whereas both differed from the Observe values in valence and arousal (P < 0.001).

### Outside-scanner behavioral measures

Further behavioral measures were obtained through the use of different scales in order to characterize emotion regulation ability, food addiction severity, impulsiveness, and inhibition/approach behavior (Table 1). Specifically, participants were administered the Emotion Regulation Questionnaire (ERQ), the Barratt Impulsiveness Scale (BIS-11), the Yale Food Addiction Scale (YFAS) and the Behavioral Inhibition System and Behavioral Activation System scales. Further details about these tasks and their psychometric properties can be found in S1 File.

### Image acquisition and preprocessing

We used a 3.0 Tesla Siemens Skyra MRI scanner, equipped with a 32-channel head coil. During acquisition, a T2*-weighted echo-planar imaging (EPI) was obtained [repetition time (TR) = 2000 ms, echo time (TE) = 37 ms, field of view (FOV) = 228 x 228 mm, 76 x 76 matrix, flip angle = 90°, 34 axial slices of 3-mm thickness, 234 scans]. A sagittal three-dimensional T1-weighted turbo-gradient-echo sequence (192 slices, TR = 2300 ms, TE = 2.07 ms, flip angle = 9°, FOV = 240 x 225, 1 mm³ voxels) was also obtained for anatomical reference.

Imaging data were transferred and processed using MATLAB version R2009a (The MathWorks Inc, Natick, MA, USA). Pre-processing was performed using statistical parametric mapping software (SPM8) (http://www.fil.ion.ucl.ac.uk/spm/). Motion correction was performed by aligning (within participant) each time series to the mean image volume using a least-squares minimization and a 6-parameter (rigid body) spatial transformation. Translation and rotation estimates (x, y, z) were required to be less than 2 mm or 2° for each participant respectively. The realigned functional sequences were subsequently coregistered to each participant’s anatomical scan, which had been previously coregistered and normalized to the SPM-T1 template. Normalization parameters were then applied to the coregistered functional images, which were then resliced to a 2mm isotropic resolution. Finally, spatial smoothing was carried out by convolution with a 3D Gaussian kernel (full width at half maximum = 8 mm).

### Statistical Analyses

#### Behavioral data analyses

We conducted independent-sample t-tests to compare the two groups on relevant variables (Table 1). Interactions between in-scanner ratings for each condition (Observe, Maintain and Regulate) and subject groups were evaluated using a 2x3 repeated-measures ANOVA analysis. Participants’ self-reported success in lowering their in-scanner negative emotion intensity was calculated by subtracting the means of participants’ Regulate ratings from the means of participants’ Maintain ratings. This value is referred to as Success and was compared between groups using an independent-sample t-test. Behavioral data were analyzed with the Statistical Package for the Social Sciences version 19 (SPSS; Chicago, IL, USA).

#### fMRI, main task effects: first-level analyses

Two contrasts of interest were defined at first-level (single-subject) analysis: (1) Maintain/Observe and (2) Regulate/Maintain. The first contrast was carried out to index brain activations associated with negative emotion generation, whereas the second contrast indexed brain activations associated with cognitive reappraisal [21,23,37,39]. Conditions were modeled for the 20 seconds that the images were on the screen and did not include instruction and rating periods. The BOLD response at each voxel was convolved with the SPM8 canonical hemodynamic response function using a 128-s high-pass filter.

#### fMRI, main task effects: second-level analyses

Between-group comparisons were conducted with two-sample t-tests using group (normal-weight vs. excess-weight) as the main factor. Keeping in mind our previously stated hypothesis regarding specific regions involved in the generation and processing of negative emotions, analyses were conducted utilizing a bilateral amygdala and insula mask for the Maintain/Observe comparison. This restriction of search regions was designed with the WFU_PICKAtlas [46].

A bilateral prefrontal region mask was created for the Regulate/Maintain contrast as this region has been implicated in the employment of reappraisal strategies [22,38]. Analyses were also conducted for this contrast using the emotion-generation mask in order to compare to what degree participants were able to shift from one task (Maintain) to another (Regulate). Additional analyses were carried out using masks generated by extracting and conjoining areas from one-sample (excess-weight and normal-weight) activations for each contrast.

Peak activations derived from the two main fMRI contrasts were extracted and entered into an SPSS data matrix to assess their relationship with behavioral measures and perform path analyses.

#### Psychophysiological interactions analysis

In order to explore the task-induced connectivity between the brain regions activated during the fMRI task, we conducted a psychophysiological interactions (PPI) analysis using SPM8 [47]. Here, we explored the impact of the two contrasts of interest (the ‘psychological’ factor) on the strength of time-course correlations between our empirically obtained ROI with all the other regions of the brain (the ‘physiological’ factor). To perform the first-level analysis (subject-level), the ROI was drawn from the set of regions showing group differences in the two contrasts from task activation analyses (Maintain/Observe and Regulate/Maintain). Since there was only one region showing group differences in both contrasts (the right anterior insula), we chose this area as our ROI. Each seed was defined as 5-mm radial spheres using MarsBaR region-of-interest toolbox [48] in MNI stereotaxic space with the coordinates derived from the peak maxima in each contrast: x = 42, y = 10, z = -12 in the case of Maintain/Observe, and x = 38, y = 18, z = -16 in the case of Regulate/Maintain.

Functional connectivity maps were estimated for the selected seed of each contrast by including our signal of interest (seed) together with the nuisance signals (CSF, white matter and global brain signal) as predictors of interest or no interest, respectively, in whole-brain linear regression analyses. A high-pass filter set at 128 seconds was used to remove low-frequency drifts of less than approximately 0.008 Hz. Contrast images were generated for each subject by estimating the regression coefficient between the seed time series and each brain voxel signal. Resulting images were then included in a two-sample t-test model for each of the contrasts (second-level) to assess for between-group effects.

#### Significance thresholding

In SPSS analyses significance threshold was set at p<0.05. In all SPM analyses (including PPI) statistical significance was determined by a combination of voxel-level and cluster-extent thresholds, using the AlphaSim algorithm as implemented in the SPM REST toolbox (http://resting-fmri.sourceforge.net/) [49]. The minimum spatial cluster extent (KE) to satisfy a family-wise error (FWE) rate correction of pFWE < .05 varied over the different contrasts. Input parameters to AlphaSim included a voxel-level probability of p<0.01, a rmm of 5, a FWHM corresponding to the actual smoothing of the data after model estimation, and a mask volume that depended on the mask being applied (ranging from 6417 to 20225 voxels for task activation analyses, and consisting on a whole-brain mask of 140825 voxels for the PPI analysis). As a result, for task activation analyses we obtained cluster-extent thresholds ranging from 44 to 158 voxels, while for the PPI analysis the thresholds were of 361 (Maintain/Observe) and 545 (Regulate/Maintain) voxels in order to satisfy a pFWE < .05.

### Emotion processing and reappraisal model

We used Pearson correlations to check whether complex relationships existed between BMI, fMRI task activation results and behavioral variables, as a preliminary step before conducting path analysis. Path analysis was carried out with maximum likelihood estimation to test both the direct and indirect predictive effect of participant BMI on these variables. This method is further explained in S1 File.

## Results

### Behavioral results

#### In-scanner behavioral measures

**Fig 2:**
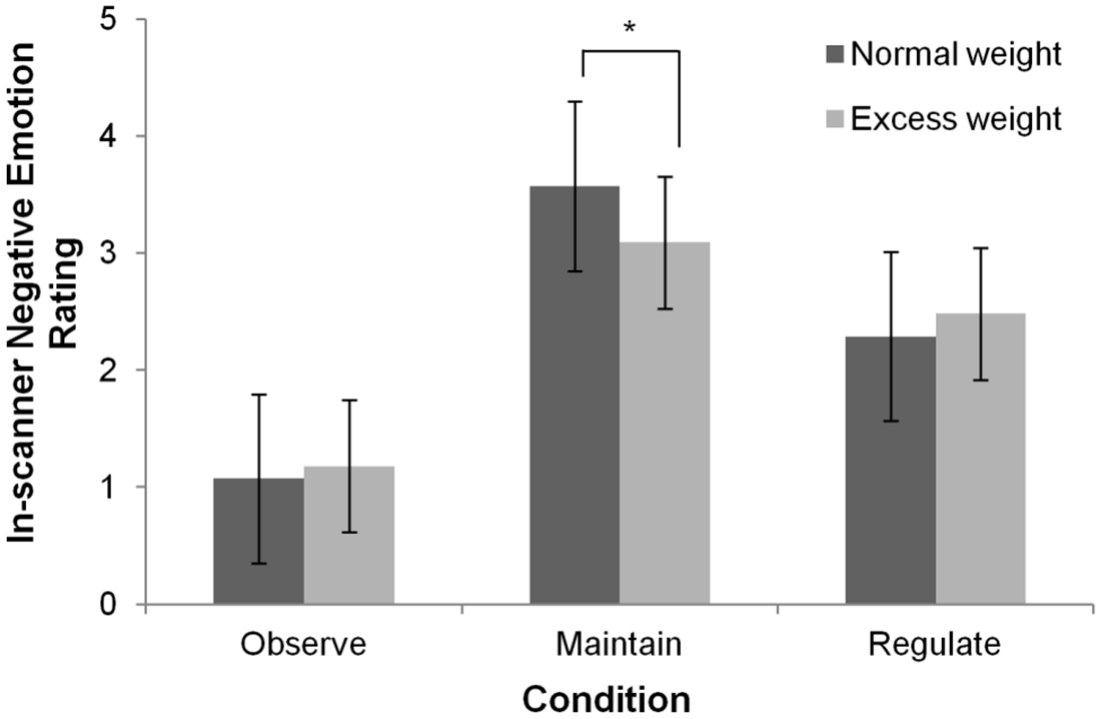
In-Scanner Negative Emotion Intensity Ratings. Mean (95% Confidence Interval) in-scanner negative emotion ratings elicited during each condition (Observe, Maintain and Regulate) (n = 28) *p<0.05.

Interactions between in-scanner negative emotion intensity ratings for each condition (Observe, Maintain and Regulate) and subject groups were evaluated using a 2x3 repeated-measures ANOVA analysis. A main effect of condition was found (F(2,52) = 165.65, p<0.001) and planned comparisons showed that Maintain differed from Observe, indicating successful negative emotion induction during this condition for both groups (Maintain>Observe: F(1,26) = 319.68, p<0.001). Moreover, planned comparisons contrasting Regulate and Maintain also showed differences between the two conditions (Maintain>Regulate F(1,26) = 56.97, p<0.001) indicating successful emotion regulation. No main effect of group was found (F(1,26) = 0.234, p>0.05). Finally, there was an interaction effect between condition and group (F(2,52) = 4.599, p = 0.014). We performed independent-sample t-tests to further interpret this interaction, and these showed no differences between the groups in Observe or Regulate ratings (p>0.05 in all cases). Interestingly, ratings for normal-weight subjects during Maintain were significantly higher than for excess-weight subjects (p = 0.043) (Table 2, Fig 2).

**Table 2:** In-scanner Negative Emotion Ratings.

In-scanner Negative	Excess weight	Normal weight	*t*
Emotion Ratings	(n = 14)	(n = 14)	*(p-value)*
**Observe**	1.17 ± 0.32	1.07 ± 0.18	**-1.098 (0.115)**
**Maintain**	3.09 ± 0.67	3.57 ± 0.52	**2.124 (0.043)**
**Regulate**	2.48 ± 0.71	2.29 ± 0.49	**-0.942 (0.355)**
**Success**	0.60 ± 0.64	1.29 ± 0.69	**2.70 (0.012)**

Data are means ±SD, 1–5 number scale (1 being “neutral” and 5 being “extremely negative”).

Using Success values, independent-sample t-tests showed that normal-weight subjects more effectively lowered their reported negative emotion intensity rating during Regulate compared to their excess-weight counterparts (p = 0.012).

#### Outside-scanner behavioral measures

Total Barratt Impulsiveness Scale (BIS-11) scores were higher for excess-weight subjects compared to normal-weight controls (p = 0.008), indicating higher levels of impulsive behavior in the former. Additional examination found that excess-weight scores in BIS-11 Attentional and Non-planning 2^nd^ order factors were greater than those of normal-weight (Table 1).

### fMRI task activations

Results regarding the common activations from both groups for the two main contrasts are provided in S1 File.

#### Maintain/Observe

**Fig 3:**
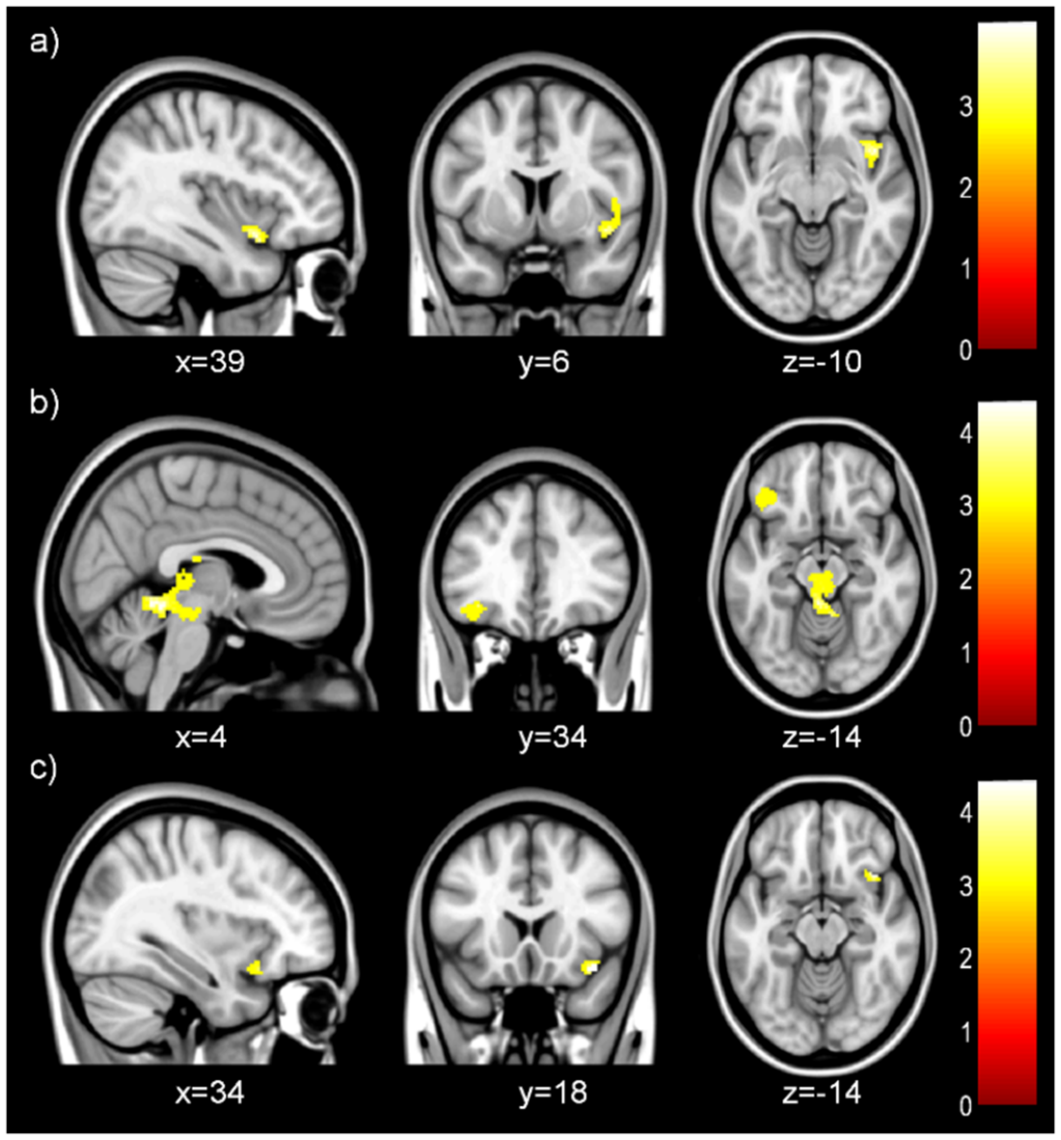
Neuroimaging Results. Between-group differences during negative emotion maintenance (Maintain/Observe) in normal-weight versus excess-weight participants. An emotion generation mask (a) and a mask consisting of combined one-sample results (b) were used. Between-group differences during negative emotion reappraisal (Regulate/Maintain) in excess-weight versus normal-weight participants (c). An emotion generation mask consisting of the amygdala and insula was used.

A direct between-groups comparison utilizing an emotion-generation mask showed that normal-weight subjects had increased activation in the right insula compared to excess-weight subjects. Comparisons using a mask generated by extracting and combining areas from one-sample activations also found greater activation in the left OFC and the bilateral cerebellar vermis in normal-weight subjects than in excess-weight subjects (Table 3, Fig 3a and 3b).

**Table 3:** Neuroimaging Results.

				MNI			
	Region	Side	(x)	(y)	(z)	k*	*t*
**Maintain>Observe**							
(Normal weight>Excess weight)							
**With Emotion-Generation Mask**	Anterior Insula	R	42	10	-14	242	3.95
**With One-Sample Mask**	Cerebellar Vermis	L/R	-2	-42	-8	1084	3.66
			4	-48	-8		3.50
	Orbitofrontal Cortex	L	-34	34	-10	174	3.58
**Regulate>Maintain**							
(Excess weight>Normal weight)							
**With Emotion- Generation Mask**	Anterior Insula	R	38	18	-16	50	4.34

Regions showing significant activations during Maintain/Observe for the normal-weight group more than the excess-weight group; and regions showing significant activations during Regulate/Maintain in excess-weight>normal-weight. An emotion-generation mask including the amygdala and the insula and a mask created including one-sample activated areas from both groups were used

* Cluster extent in voxels.

#### Regulate/Maintain

No between-group differences using the emotion-regulation mask passed significance threshold. However, a direct between-groups comparison using the emotion-generation mask showed that excess-weight subjects had greater activation in the right anterior insula compared to normal-weight controls (Table 3, Fig 3c).

### PPI Analyses

#### Seed selection

Task-induced functional connectivity analyses were performed to explore whether specific regions showing significant between-group differences in the main task contrasts were abnormally connected with other regions of the brain. Among the clusters showing significant between-group differences, we selected the right insula, which showed greater activation in normal-weight versus excess-weight participants during Maintain/Observe, and greater activation in excess-weight versus normal-weight subjects during Regulate/Maintain (see Table 3 and Fig 3).

#### Group differences in Maintain/Observe

No significant between-group differences were found for this contrast.

#### Group differences in Regulate/Maintain

**Fig 4:**
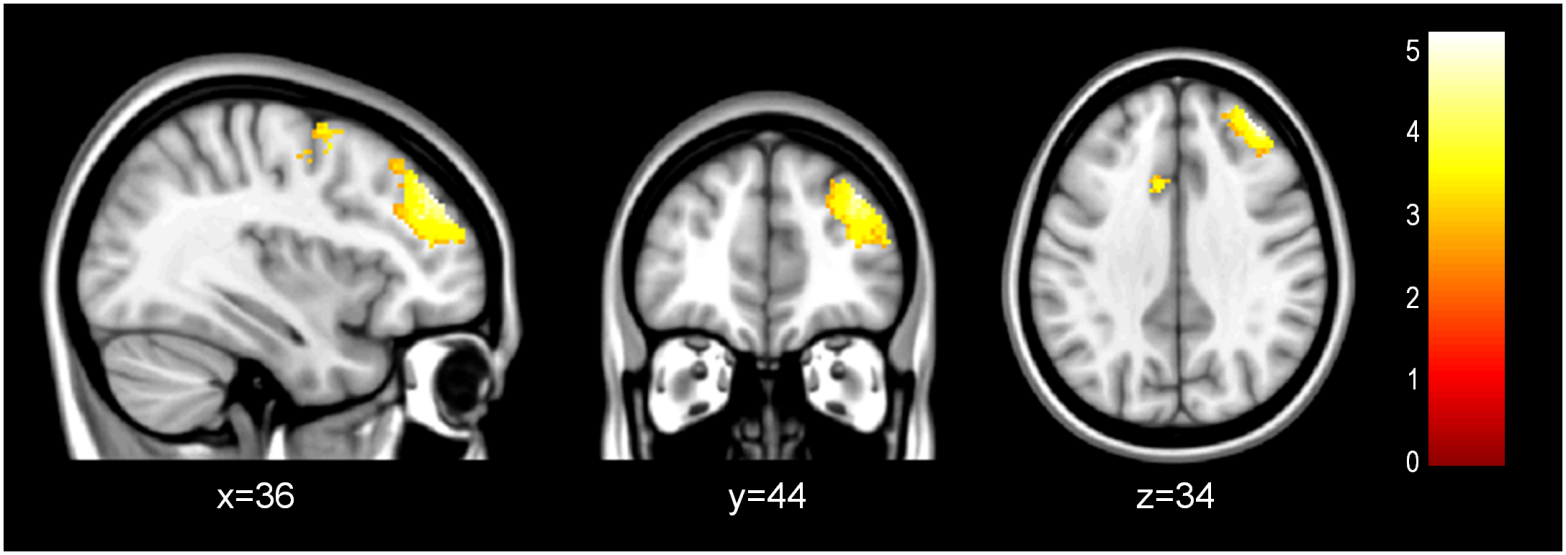
PPI Results. Regions showing different patterns of connectivity with the right insula seed between normal-weight individuals compared to excess-weight participants during reappraisal (Regulate>Maintain), with normal-weight subjects presenting a significant anticorrelation between the right insula and the PFC regions shown.

In normal-weight participants, as compared to those with excess-weight, the right insula seed showed significantly increased negative functional coupling with the right dlPFC, and the bilateral dmPFC (Table 4, Fig 4).

**Table 4:** PPI Results.

				MNI			
	Region	Side	(x)	(y)	(z)	k*	*t*
**Regulate>Maintain**							
**(Normal weight<Excess weight)**							
	dlPFC	R	36	44	34	882	**5.18**
	dmPFC	L	-20	-12	42	1416	**4.73**
	dmPFC	R	28	-6	46	1031	**3.82**

Regions showing a significant anticorrelation with the right insula seed during Regulate/Maintain in the normal-weight group compared to the excess-weight group at the whole-brain level

* Cluster extent in voxels.

### Emotion processing and reappraisal model

Significant correlations between BMI, right insula activations during Maintain/Observe and Regulate/Maintain, BIS-11 2nd Order Attentional scores, and in-scanner Maintain and Success ratings suggested the existence of complex pathways between them and were further tested with path analysis. The whole set of correlations used for the path analysis can be found in S1 File.

**Fig 5:**
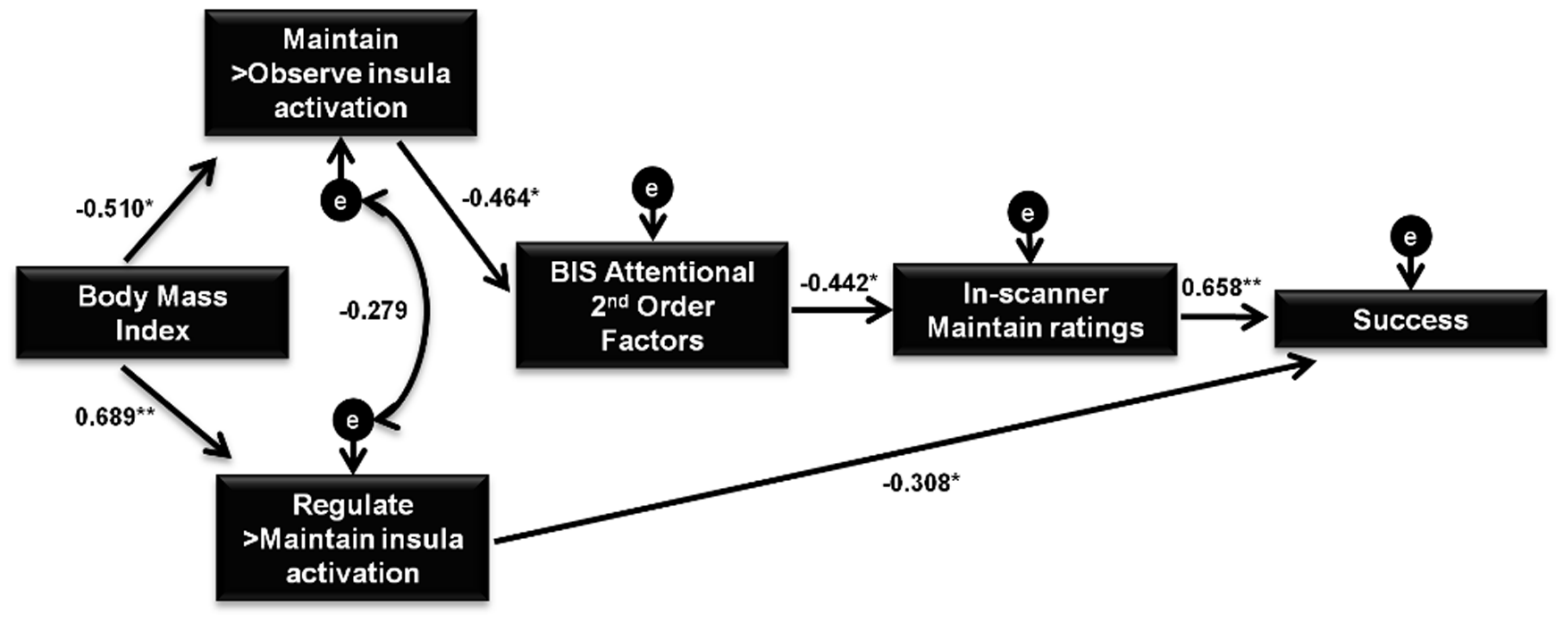
Final Path Analysis Model. Model 3 was the best representation of the observed relationship between variables and examined the complex associations between Body Mass Index (BMI), neuroimaging results and behavioral data. BIS-11 = Barratt Impulsiveness Scale; Standardized regression weights for direct effects are shown *p<0.05 **p<0.001.

Different models were tested (see S1 File) and the final model obtained is summarized in Fig 5, and was considered to best represent the observed relationships between the variables. This model provided good fit and all direct effects were highly significant. Specifically, increased BMI was directly associated with decreased right insula activity during Maintain/Observe and with heightened right insula activity during Regulate/Maintain. Accordingly, increased right insula activity during Maintain/Observe was found to directly predict lower BIS-11 2nd Order Attentional scores, and these in turn predicted higher in-scanner negative emotion ratings during Maintain. Subsequently, higher in-scanner Maintain ratings directly predicted greater Success scores. Finally, increased Regulate/Maintain insula activity was associated with lower Success ratings.

The exploration of the squared multiple correlations showed that Model 3 accounted for 26% of the variance of right insula activation during Maintain/Observe, 47% of right insula activation during Regulate/Maintain, 21% of BIS-11 2nd Order Attentional scores and 19% and 57% of in-scanner Maintain negative emotion ratings and Success, respectively.

Further information regarding model fitting of path analysis and the indirect associations of the final model can be found in S1 File.

## Discussion

In this study, we aimed to compare the neural substrates of negative emotion processing and emotion regulation in people with excess weight using fMRI. Our main finding showed that excess-weight subjects displayed decreased insula activation when experiencing negative emotions and increased activation when instructed to regulate these emotions using cognitive reappraisal. This is in keeping with our hypothesis that participants with excess weight would display persistently heightened activation in emotion-generation brain regions during reappraisal. Contrary to our original hypothesis, participants with excess weight did not show hyperactivation in the insula and amygdala during negative emotion experience nor did we find hypoactivation in prefrontal regions during reappraisal. Notwithstanding, PPI connectivity analyses found that excess-weight participants had reduced negative connectivity between the right anterior insula and prefrontal regions during cognitive reappraisal compared to normal-weight controls. Decreased activation in the OFC and cerebellum during negative emotion experience was also found in excess-weight subjects.

The somatic marker hypothesis (SMH) argues for a mechanism by which emotional processes can steer (or bias) behavior via physiological markers [50]. The key idea of this hypothesis is that somatic marker signals influence the higher-order processing of affective stimuli and choices, at multiple levels of operation, some of which occur overtly (consciously) and some of which occur covertly (non-consciously). This theory uploads that “cognitive operations, regardless of their content, depend on support processes such as attention, working memory and emotion” and that body states serve as a form of dispositional knowledge to guide reasoning and decision making [51]. Our results suggest that excess weight may be linked to a decreased ability to allocate attentional resources to engage physiological “feedback/warning” markers when facing negative-emotion-inducing stimuli [52]. This conclusion is supported by the findings of our path analysis, which found that increased BMI was directly associated with decreased right insula activity during the Maintain-Observe contrast and that this decrease in right insula activation predicted poor attentional impulsiveness performance on the BIS-11 and lower in-scanner negative emotion ratings. The insula is ascribed to a vast number of functional properties, including gustatory processing [53], and serves as a crossroads for integrating homeostatic feedback with expected outcomes [54]. By acting as a “preferential clearinghouse” for such interoceptive markers, disrupted insula activation in excess-weight participants could lead to an adverse physiological state that adjusts the tendency to direct focal attentional resources in one direction over another. This is in line with the findings of our previous studies showing that excess weight is associated with insula dysfunctions relevant to the perception of interoceptive signals [55] and the anticipation of risky choices [56].

When instructed to use emotion regulation, our study found that excess weight subjects did not lower right anterior insula activity to the same extent as normal-weight control subjects. The anterior insula is known to be involved in the secondary processing of emotional experience through the integration of interoceptive signals with external context [28,54], a key aspect of successful cognitive reappraisal [38]. This is suggestive of the notion that excess-weight participants may be less able to engage the optimal brain circuitry needed to exert effective down-regulation of emotion-generation regions as high BMI correlated with increased activity in the right anterior insula during emotion regulation and consequently predicted poorer reappraisal performance. This correlation dovetails with another study using real-time fMRI (rtfMRI) which found that participants with an increased BOLD signal in the insula rated aversive pictures more negatively compared to those having a decreased signal [57]. Moreover, our PPI analyses found reduced negative connectivity between the right anterior insula and the dlPFC and the dmPFC in participants with excess weight during Regulate/Maintain. The dmPFC and the dlPFC are ascribed to the monitoring of emotional states and the use of working memory to model appraisals, respectively [23,24]. This finding indicates that excess-weight participants may be unable to exert effective regulation of emotional responses via prefrontal regions when facing negative stimuli.

We also found that excess-weight subjects had decreased activation in the OFC when experiencing negative emotions compared to normal-weight subjects. The OFC is critically involved in the evaluation of affective feedback and related cognitive control functions, such as value-based decision-making [58]. Decreased OFC activation in this group when experiencing emotions could be indicative of abnormal evaluation of negative stimuli and hence could hamper efforts to restrain unhealthy food choices [9]. Likewise, excess-weight participants displayed lower bilateral cerebellar vermis activation during the Maintain-Observe contrast compared to normal-weight controls. fMRI evidence suggests that the cerebellar vermis serves as a node in the corticolimbic network and is involved in detecting, integrating and filtering emotional information [59]. As patients with lesions to this specific area of the cerebellum exhibit a flattening of affect, excess-weight participants’ deceased activation of this region when exposed to negative images could be responsible for their diminished in-scanner behavioral response during Maintain.

There are several limitations to the present study. Firstly our sample size was rather modest and all data were collected from young, college-educated individuals. Future studies should aim to include participants from a wider range of different ages and social backgrounds. Secondly, our study failed to find differences in neural activation in prefrontal regions either within or between groups, at difference with previous studies [22,38], though we did find differences regarding task-induced prefronto-limbic connectivity. We propose that this discrepancy is related to the fact that prefrontal regions continue to undergo development far into young adulthood [60], and thus it is possible that this sample of young adults did not utilize frontal control over subcortical regions in the same way a more heterogeneous, older sample would have [61]. Gaining a better understanding of prefrontal development is of importance as other studies have found that adolescents display hypo-responsivity of inhibitory regions while anticipating intake of sugary drinks [62]. Finally, possible differentiating activation patterns among subjects with binge eating disorder (BED) were not examined. This distinction warrants further research since emotion-regulation deficits are a widely used explanation for the development and maintenance of BED [63].

Taken together, these results demonstrate for the first time the differential patterns of brain activation during emotion generation and regulation in individuals with excess weight: these patterns are suggestive of poorer perception of interoceptive signals when experiencing emotions and less effective down-regulation of the emotion-generation network during reappraisal. This study adds neurobiological support to the abundance of evidence demonstrating that difficulties in emotion regulation influence eating and lead to increased food intake [32,63]. Our results reinforce the pertinence of developing novel treatment interventions aimed at improving emotion regulation strategies, impulsiveness and focal attention in individuals with excess weight or at risk of becoming overweight [64,65].

## Supporting Information

S1 File Supporting Information. (DOCX)
