# Supplementary material for: Emotion Regulation and Excess Weight: Impaired Affective Processing Characterized by Dysfunctional Insula Activation and Connectivity
Source: PLoS One. 2016 Mar 22;11(3):e0152150. doi: 10.1371/journal.pone.0152150 (PMC4803189; doi:10.1371/journal.pone.0152150)
Supplement: S1 File — (DOCX) [file pone.0152150.s001.docx]

**Supporting Information**

**Emotion regulation and excess weight: impaired affective processing characterized by dysfunctional insula activation**

Trevor Steward^a,b,f¶^, Maria Picó-Pérez^a,b¶^, Fernanda Mata^c^, Ignacio Martínez-Zalacaín^a^, Marta Cano^a,b^, Oren Contreras-Rodríguez^a,d^, Fernando Fernández-Aranda^a,b,f^, Murat Yucel^c^, Carles Soriano-Mas^a,d,e#^, Antonio Verdejo-García^c^

^a^Department of Psychiatry, Bellvitge University Hospital-IDIBELL, Barcelona, Spain; ^b^Department of Clinical Sciences, School of Medicine, University of Barcelona, Spain; ^c^School of Psychological Sciences and Monash Institute of Cognitive and Clinical Neurosciences, Monash University, Melbourne, Australia;

^d^CIBER Salud Mental (CIBERsam), Instituto Salud Carlos III (ISCIII), Barcelona, Spain

^e^Department of Psychobiology and Methodology in Health Sciences, Universitat Autònoma de Barcelona, Spain

^f^CIBER Fisiopatología de la Obesidad y Nutrición (CIBERObn), Instituto Salud Carlos III (ISCIII), Barcelona, Spain

^¶^ Equal contribution

^*^ Corresponding author

Carles Soriano-Mas, PhD,

Email address: csoriano@idibell.catcsoriano@idibell.cat

**Methods**

**Outside-scanner behavioral measures**

The Emotion Regulation Questionnaire (ERQ) is designed to assess individual differences in the habitual use of two emotion regulation strategies: cognitive reappraisal and expressive suppression [1]. Cognitive reappraisal is defined as an active, cognitive strategy where situations are reappraised so that their emotional impact is lessened or strengthened, whereas expressive suppression is defined as a response-focused strategy where behavioral reactions or emotional expressions are concealed by means of restraining or inhibiting external facial or bodily signs of emotion. This 10-item questionnaire is commonplace in studies of ER and has presented good psychometric properties. It has demonstrated adequate to good internal consistency and temporal stability [2,3] as well as good convergent and discriminant validity [4].

The Barratt Impulsiveness Scale (BIS-11) [5] is a questionnaire designed to assess the personality/behavioral construct of impulsiveness. It is one of the most widely used instruments for the assessment of impulsiveness and includes 30 items that are scored to yield six first-order factors (attention, motor, self-control, cognitive complexity, perseverance, and cognitive instability impulsiveness) and three second-order factors (attentional, motor, and non-planning impulsiveness). This scale has reported internal consistency coefficients ranging from 0.79 to 0.83 for separate populations of university students, substance-abuse patients, general psychiatric patients, and prison inmates [5].

The Yale Food Addiction Scale (YFAS) is a 25-point questionnaire, based on DSM-IV codes for substance dependence criteria, to assess food addiction in individuals [6]. It has been developed to identify those who are most likely to exhibit markers of substance dependence with the consumption of high fat/high sugar foods and assesses clinically significant impairment or distress from eating. The YFAS has exhibited adequate internal reliability, and showed good convergent validity with measures of similar constructs and good discriminant validity relative to related but dissimilar constructs [7].

The Behavioral Inhibition System and Behavioral Activation System scales are designed to assess dispositional sensitivity to the behavioral inhibition system and the behavioral activation or behavioral approach system [8]. The Behavioral Activation System is related to motivations to seek out positive experiences and is commonly referred to as approach motivation, whereas the Behavioral Inhibition System is sensitive to signs of fear-inducing stimuli and is commonly referred to as avoidance motivation. This 20-item questionnaire uses a 4-point scale and examines individual differences in the sensitivity of these systems. This questionnaire has displayed satisfactory psychometric properties and all the subscales have been reported to have satisfactory internal consistencies, α’s ranging from .66 to .76, and two-month test–retest reliabilities, r’s ranging from .59 to .69 [8].

**Emotion processing and reappraisal model**

Path analysis is a method used to estimate a set of simultaneous regression equations to explore how a group of variables interrelate in complex patterns. Three types of effects can be distinguished: (1) direct effects, which demonstrate the direct association of one variable with another; (2) indirect effects, which indicate the indirect association of one variable with another via other variables in the model; (3) total effects, which estimate the addition of direct and indirect effects. The first step of this process consists of testing an initial just-identified or saturated model in which the number of free parameters equals the number of known values (i.e., a model with zero degrees of freedom). The following steps consist of performing a trimming of this saturated model by retaining the significant or trend-level significant associations and excluding non-significant paths. [9]

Path analysis was conducted by means of AMOS 18.0 software and the significance of indirect effects was tested using a bootstrapping method [10]. Model fit was assessed using the following indices: i) Chi-square statistic (X2), which acts as a measure of misfit where non-significant values imply a good fit of the model to the data; ii) goodness of fit index (GFI), which indicates the proportion of the variance in the sample variance-covariance matrix that is accounted for by the model (expected to be greater than 0.95); iii) root mean square error of approximation (RMSEA), which estimates the lack of fit compared to the saturated model (where values up to 0.05 indicate a good fit); and iv) comparative fit index (CFI), which compares the fit of the proposed model to a null model where none of the variables are related between each other (with values greater than 0.95 indicating a good fit).

**Results**

**Imaging Results**

**Common Task Activations**

Widespread activations were found in bilateral posterior sensory regions, the thalamus, the insula, the amygdala, the cerebellum and regions of the midbrain and prefrontal cortex (Fig S1a) for both groups during Maintain>Observe.

On the other hand, activations were found in the left precentral gyrus in both groups during Regulate>Maintain (Fig S1b), as well as deactivations in the left posterior insula and the cuneus (Fig S1c).

**
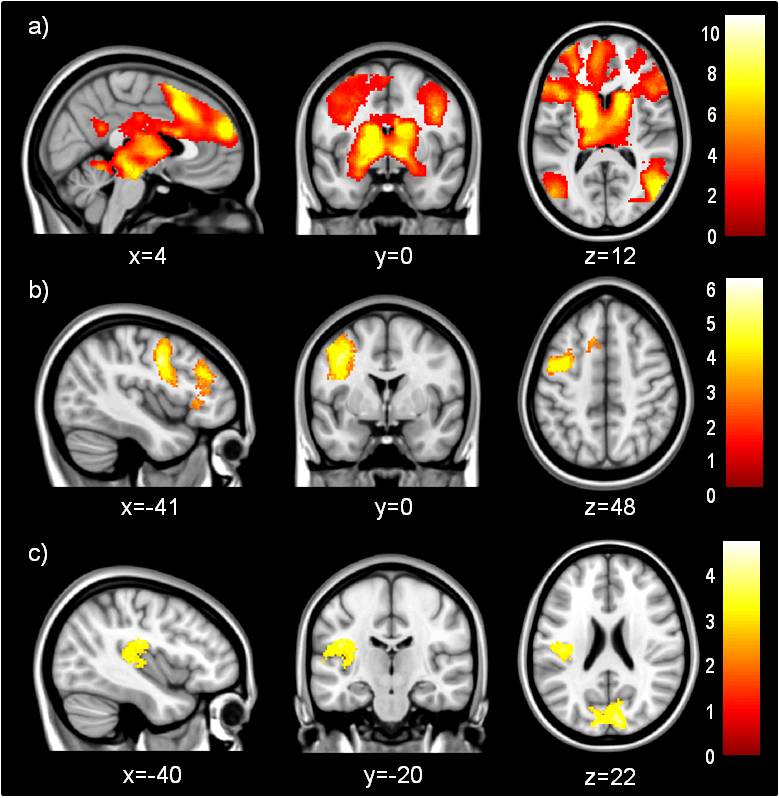
**

**Figure S1.** Areas showing increased activation in both normal-weight and excess-weight participants during (a) negative emotion maintenance (Maintain>Observe) and (b) reappraisal (Regulate>Maintain), and (c) areas showing decreased activation during reappraisal (Maintain>Regulate).

**Path analysis**

In order to not distort path analysis results, data from one participant was excluded due to outlying imaging results during Maintain>Observe.

|  | **BMI** | **Maintain >Observe insula activation** | **Regulate >Maintain insula activation** | **BIS-11 Attentional 2^nd^ Order Factor** | **In-scanner Maintain ratings** | **Success** |
| --- | --- | --- | --- | --- | --- | --- |
| **BMI** | 1 |  |  |  |  |  |
| **Maintain>Observe insula activation** | -0.510^**^ | 1 |  |  |  |  |
| **Regulate>Maintain insula activation** | 0.689^**^ | -0.525^**^ | 1 |  |  |  |
| **BIS-11 Attentional 2^nd^ Order Factor** | 0.408^*^ | -0.464^*^ | 0.363 | 1 |  |  |
| **In-scanner Maintain ratings** | -0.287 | 0.143 | -0.182 | -0.442^*^ | 1 |  |
| **Success** | -0.401^*^ | 0.340 | -0.421^*^ | -0.398^*^ | 0.703^**^ | 1 |

**Table S1.** Pearson’s correlations between Body Mass Index (BMI), right insula activations during Maintain>Observe and Regulate>Maintain, Barratt Impulsiveness Scale (BIS-11) Attentional 2nd Order Factor scores and in-scanner Maintain and Success ratings *Significant (p<0.05) **Significant (p<0.01).

**Model trimming**

Model 1 (Fig S2) provided good fit statistics (Table S2) and the direction of the associations was in agreement with BMI having a predictive effect on imaging results and behavioral outcomes. However, a lack of statistical significance of the regression weights that connected BMI, behavioral and imaging results directly with in-scanner reappraisal Success ratings showed that the model was overcomplicated. Therefore, we analyzed a second model (Model 2) that excluded these non-significant associations.

**Figure S2.** Model 1, which explored all significant correlations between Body Mass Index (BMI), behavioral and imaging variables. Lack of statistical significance of some of the explored effects suggest that the model may be overcomplicated. Standardized regression weights for direct effects are shown *p<0.05 **p<0.001.

| **Values indicative of good fit** | X^2^ | p value | df | GFI >0.95 | RMSEA <0.05 | CFI >0.95 |
| --- | --- | --- | --- | --- | --- | --- |
| Model 1 | 1.833 | .872 | 5 | 0.978 | 0 | 1 |
| Model 2 | 3.187 | .922 | 8 | 0.961 | 0 | 1 |
| Model 3 | 3.187 | .922 | 8 | 0.961 | 0 | 1 |

**Table S2.** Model fit indices for tested models: X^2^ chi-square statistic, df degrees of freedom, GFI goodness of fit index, RMSEA root mean square error of approximation, CFI comparative fit index.

Model 2 showed an improved fit to the data. Still, the regression weight directly linking the right insula peak for Maintain>Observe and the right insula peak for Regulate>Maintain was non-significant. Consequently, we analyzed a more parsimonious model that excluded this non-significant association.

**Indirect associations between variables**

The best fitting model, model 3, showed that increased BMI was indirectly associated with higher BIS-11 2nd order Attentional scores (standardized indirect effect = 0.237, p=0.010), decreased in-scanner negative emotion ratings during Maintain (standardized indirect effect = -0.105, p=0.010) and decreased Success scores (standardized indirect effect = -0.281, p=0.026). Increased right insula activation during Maintain>Observe indirectly predicted higher in-scanner negative emotion ratings during Maintain (standardized indirect effect = 0.205, p=0.010) and also indirectly predicted increased Success scores (standardized indirect effect = 0.135, p=0.010). Lastly, higher BIS-11 2^nd^ order Attentional scores showed an indirect, negative effect on Success scores (standardized indirect effect = -0.291, p=0.010).

**References**

1. Gross JJ, John OP (2003) Individual differences in two emotion regulation processes: implications for affect, relationships, and well-being. J Pers Soc Psychol 85: 348–362.
2. Sala M, Molina P, Abler B (2012) Measurement invariance of the Emotion Regulation Questionnaire (ERQ). A cross-national validity study. European Journal of Developmental Psychology 9: 6.
3. Batistoni S, Ordonez T (2013) Emotional Regulation Questionnaire (ERQ): psychometric indicators and affective relations in an elderly sample. Psicol Reflex Crit 26: 10-18.
4. Ioannidis CA, Siegling AB (2015) Criterion and incremental validity of the emotion regulation questionnaire. Front Psychol 6: 247.
5. Patton JH, Stanford MS, Barratt ES (1995) Factor Structure of the Barratt Impulsiveness Scale. Journal of Clinical Psychology 768–774.
6. Gearhardt AN, Corbin WR, Brownell KD (2009) Food Addiction An Examination of the Diagnostic Criteria for Dependence. J Addict Med 3: 42–45.
7. Gearhardt AN, Corbin WR, Brownell KD (2009) Preliminary validation of the Yale Food Addiction Scale. Appetite 52: 430–436.
8. Carver CS, White TL (1994) Behavioral inhibition, behavioral activation, and affective responses to impending reward and punishment: The BIS/BAS Scales. J Pers Soc Psychol 67: 319–333.
9. Byrne BM. Structural Equation Modeling with AMOS. 2nd ed. New York: 2010.
10. Shrout PE, Bolger N (2002) Mediation in experimental and nonexperimental studies: new procedures and recommendations. Psychol Methods 7: 422-445.
